# Increased Numbers of Enteric Glial Cells in the Peyer’s Patches and Enhanced Intestinal Permeability by Glial Cell Mediators in Patients with Ileal Crohn’s Disease

**DOI:** 10.3390/cells11030335

**Published:** 2022-01-20

**Authors:** Olga Biskou, Felipe Meira de-Faria, Susanna M. Walter, Martin E. Winberg, Staffan Haapaniemi, Pär Myrelid, Johan D. Söderholm, Åsa V. Keita

**Affiliations:** 1 Department of Biomedical and Clinical Sciences, Linköping University, 58185 Linköping, Sweden; olga.biskou@liu.se (O.B.); felipe.meira.de.faria@liu.se (F.M.d.-F.); susanna.walter@liu.se (S.M.W.); martin.winberg.tinnerfelt@liu.se (M.E.W.); par.myrelid@liu.se (P.M.); johan.d.soderholm@liu.se (J.D.S.); 2 Department of Gastroenterology, Linköping University, 58185 Linköping, Sweden; 3 Department of Surgery, Vrinnevi Hospital, 60182 Norrköping, Sweden; Staffan.Haapaniemi@regionostergotland.se; 4 Department of Surgery, Linköping University Hospital, 58185 Linköping, Sweden

**Keywords:** neuro-immune interactions, gut inflammation, enteric nervous system, follicle-associated epithelium

## Abstract

Enteric glial cells (EGC) are known to regulate gastrointestinal functions; however, their role in Crohn’s disease (CD) is elusive. Microscopic erosions over the ileal Peyer’s patches are early signs of CD. The aim of this work was to assess the localization of EGC in the follicle and interfollicular region of the Peyer’s patches and in the lamina propria and study the effects of EGC mediators on barrier function in CD patients and non-inflammatory bowel disease (non-IBD) controls. EGC markers, glial fibrillary acidic protein (GFAP), and S100 calcium-binding protein β (S100β) were quantified by immunofluorescence and Western blotting. Both markers showed significantly more EGC in the Peyer’s patches and lamina propria of CD patients compared to the non-IBD controls. In CD patients there were significantly more EGC in Peyer’s patches compared to lamina propria, while the opposite pattern was seen in controls. Barrier function studies using Ussing chambers showed increased paracellular permeability by EGC mediators in CD patients, whereas permeability decreased by the mediators in controls. We show the accumulation of EGC in Peyer’s patches of CD patients. Moreover, EGC mediators induced barrier dysfunction in CD patients. Thus, EGC might have harmful impacts on ongoing inflammation and contribute to the pathophysiology of the disease.

## 1. Introduction

Crohn’s disease (CD) is an inflammatory bowel disease (IBD) with an unknown etiology; however, it is well established that genetic [1,2], environmental [3], microbial [4], and immunological factors [5] contribute to the disease pathogenesis. One of the first observable signs of ileal CD are aphthoid lesions of the follicle-associated epithelium (FAE) covering the Peyer’s patches [6]. The Peyer’s patches are important for immune responses and have been associated with CD pathogenesis [6,7,8]. Peyer’s patches are dome-like structures, consisting of a follicle with a B-cell germinal center surrounded by a T-cell interfollicular region (IFR). Within the Peyer’s patches, there are a variety of immune cells with the region between the FAE and the follicle, the subepithelial dome, being rich in dendritic cells and macrophages [9]. We recently showed [10] a higher number of mast cells and an up-regulation of mast cells expressing receptors for vasoactive intestinal polypeptide (VIP) in the Peyer’s patches and IFR of patients with CD compared to non-IBD controls. This suggests more neuro-immune input at the Peyer’s patches and the IFR in CD patients, which could imply an important regulatory role for this region.

The FAE differs from the surrounding villus epithelium (VE) covering the lamina propria, as this epithelium is specialized for sampling and transport of antigen into the underlying tissue [9]. In addition, the VE adjacent to the FAE, next to the IFR, was shown to differ from the VE situated further away from the Peyer’s patches [11]. We previously showed an enhanced transport of antigens and bacteria through the FAE compared to the surrounding regular VE [12]. Moreover, we showed an enhanced bacterial uptake in the FAE of CD patients compared to non-IBD controls [13]. The mechanism underlying the impaired barrier function of Peyer’s patches in CD is not fully understood, but neuro-immune interactions involving mast cells and eosinophils have been implicated in the disturbed barrier function [14]. The integrity of the barrier is also known to be regulated by the enteric nervous system (ENS) and the enteric glial cells (EGC) [14,15]. EGC help to maintain the integrity of the barrier by promoting the proliferation and differentiation of the intestinal epithelial cells, alongside the expression of genes responsible for the maintenance of the barrier. The EGC communicate with neurons of the ENS, seemingly through ATP, and react to both intrinsic and extrinsic neuronal stimuli through a variety of mediators [16]. Growth factors secreted by the EGC, such as glial-derived neurotrophic factor (GDNF) and S-nitroglutathione (GSNO), are shown, under normal conditions, to preserve the mucosal integrity, by up-regulating the production of barrier-forming tight junction proteins [17]. Furthermore, GDNF is reported to have an anti-inflammatory role in preventing the apoptosis of EGC [18] and by reducing the levels of pro-inflammatory cytokines [15].

Although the evidence for the role of EGC in the regulation of the intestinal barrier is growing [17], there are limited studies about EGC in CD, and to our knowledge, there are no reports on the role of EGC in Peyer’s patches. Therefore, we aimed to study the distribution of EGC in the Peyer’s patches and the surrounding lamina propria, in patients with CD and non-IBD controls. Furthermore, we studied the effect of EGC mediators on the integrity of the epithelial barrier in patients with CD and non-IBD controls.

## 2. Materials and Methods

### 2.1. Patients and Sample Collection

Microscopically non-inflamed specimens from the terminal ileum next to the ileocecal valve, or in patients who underwent a previous resection from the neo-terminal ileum, were taken during surgery from a total of 20 patients with CD, median age 43 years (range 17–63, 12 men), at the University Hospital of Linköping. Patient characteristics (anti-inflammatory medication, primary/recurrent surgery, indication for surgery, Montreal classification, and pre-operative plasma-C-reactive protein (an acute phase protein corresponding to inflammation and commonly used as a marker of enteric inflammation in CD)) are given in Table 1. As non-IBD controls, macro-and microscopically normal ileal specimens were received from 24 patients, median age 73 years (range 52–82, 10 men), during surgery for colonic cancer at the University Hospital of Linköping or Vrinnevi Hospital, Norrköping. The patients had no generalized disease and none had received preoperative chemo- or radiotherapy. The study was approved by the Committee of Human Ethics, Linköping (ethical number 02–154, 9 April 2002), and tissues were collected between 2016–2020. All subjects gave their written informed consent following the Helsinki declaration before the study was initiated.

Directly after dissection during surgery, intestinal tissue was put in ice-cold oxygenated Krebs buffer (115 mM NaCl, 1.25 mM CaCl_2_, 1.2 mM MgCl_2_, 2 mM KH_2_PO_4_, and 25 mM NaHCO_3_, pH 7.35) and transported to the laboratory. The muscularis propria and myenteric plexus were stripped off the mucosa and segments of Peyer’s patches and lamina propria were microscopically identified as previously described [12]. Removing of these layers is necessary for the barrier function experiments to enable measurements of the passage of the markers. Segments were either snap-frozen for Western blotting, fixed in 4% paraformaldehyde in PBS for immunofluorescence staining, or directly mounted in Ussing chambers for barrier function studies.

### 2.2. Quantification of GFAP and S100β by Immunofluorescence

**Figure 1:**
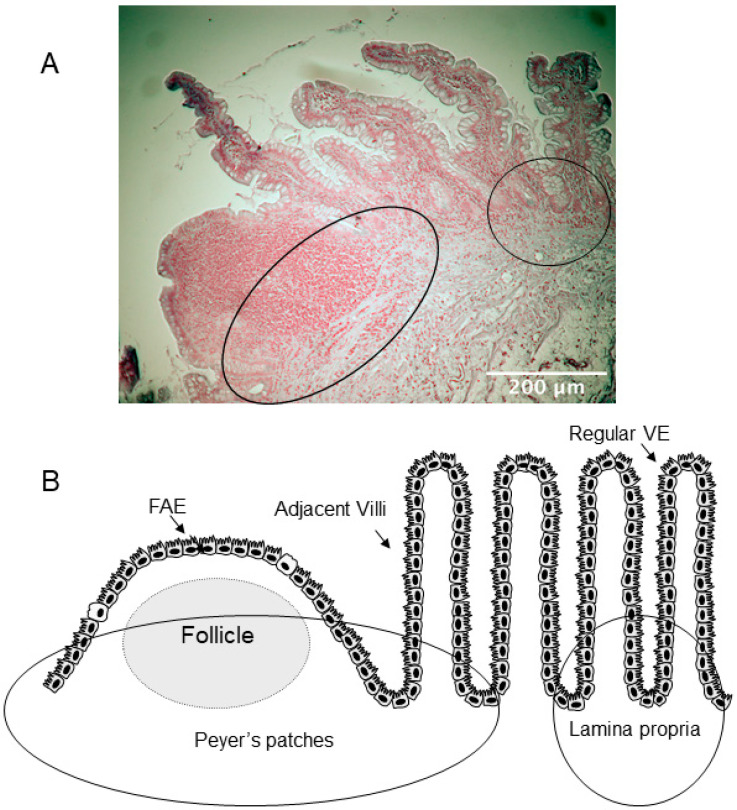
Descriptive overview of the two areas analyzed for enteric glial cells (EGC) distribution. (**A**) Representative hematoxylin-stained image used for analysis. Circles represent the two areas analyzed; Peyer’s patches, covered by the follicle-associated epithelium (FAE), and lamina propria covered by regular villus epithelium (VE). Scale bar 200 μm. (**B**) Schematic representation of the two areas. The left circle represents the area defined as Peyer’s patches, which includes the follicle, the interfollicular region, and the adjacent villi. The circle to the right defines the area representing lamina propria. In the image, the circle identifying lamina propria is situated close to the Peyer’s patches, though, only areas of lamina propria that were situated at least six villi away from the Peyer’s patches were analyzed.

Tissue segments from 12 CD patients, median age 40 years (range 20–63, 6 men), and 12 non-IBD controls, median age 73 years (range 52–80, 6 men), were embedded in paraffin and sectioned at 5 µm. Once sectioned, samples were incubated at 60 °C for 2 h. Deparaffinization and rehydration was performed by routine procedure involving incubating the sections for 5 min in Histoclear (Histolab, Gothenburg, Sweden), followed by incubation in 99.5% ethanol, 95% ethanol, 70% ethanol, and finally in H2O. The antigen retrieval was performed by boiling in citrate buffer (10 mM tri-sodium citrate dihydrate in H2O, pH 6, 0.5% Tween 20). Sections were allowed to cool to room temperature and then permeabilized using PBS supplemented with 0.1% Triton X for 10 min, followed by blocking with 1% BSA in PBS-0.5% Tween containing 300 mM glycine, for 30 min. Sections were individually stained for rabbit-anti-GFAP (1:500; Dako Cytomation, Glostrup, Denmark) and mouse-anti-S100β (1:250; Invitrogen, Gothenburg, Sweden) followed by incubation with Alexa Fluor 594-conjugated secondary antibodies (1:2000; Invitrogen) and mounting with Prolong^®^ Gold DAPI (Thermo Fisher, Stockholm, Sweden), as previously described [19]. The specificity of the secondary antibody was obtained by omitting primary antibodies. The mucosal layer was evaluated at magnification 60× and cells stained for GFAP and S100β, respectively, were quantified in 2–3 images/section of lamina propria and 2–3 images/section in the Peyer’s patches, according to Figure 1B. The evaluated area defined as Peyer’s patches included the follicle and the IFR, but also the adjacent villi, since this region has shown to differ from the villi situated further away from the Peyer’s patches [11]. For evaluation of EGC in the lamina propria, only areas of lamina propria that were situated at least six villi away from the Peyer’s patches were analyzed. Quantification was done in a blinded fashion by two independent researchers using a Nikon E800 fluorescence microscope connected to software NIS elements (Nikon Instruments Inc., Tokyo, Japan).

### 2.3. Western Blotting for GFAP and S100β

Protein was extracted from frozen ileal tissue of Peyer’s patches and lamina propria from 12 patients with CD, median age 44 years (range 21–52, 7 men), and 12 non-IBD controls, median age 71 years (range 54–80, 6 men), as described previously [13], followed by Western blotting. Protein was extracted as previous described [13] using RIPA buffer (Thermo Fisher, Stockholm, Sweden). Protein, 20 μg per sample, was run on a 16%, or a 4–20% Tris-Glycine SDS-gel (Thermo Fisher, Stockholm, Sweden). Proteins were transferred to nitrocellulose membrane (Amersham, Darmstadt, Germany), in Tris-Glycine buffer (Thermo Fisher, Stockholm, Sweden) supplemented with 20% ethanol. Following the transfer, membranes were blocked with 5% purified milk protein (Bio-Rad, Solna, Sweden) for 1 h at room temperature. Membranes were incubated overnight at 4 °C with rabbit polyclonal antibody anti-GFAP (1:5000; Dako Cytomation, Stockholm, Sweden), rabbit monoclonal anti-S100β antibody (1:1000; Abcam, Cambridge, UK), mouse monoclonal anti-β-actin antibody (1:10,000; Cell Signaling, BioNordika, Solna, Sweden), in TBS pH 7.6, 5% *w*/*v* BSA and 0.05% *v*/*v* Tween 20. Membranes were washed and incubated with Alexa Fluor 790-conjugated goat polyclonal-anti-mouse (1:20,000; Thermo Fisher, Stockholm, Sweden) and Alexa Fluor 680-conjugated goat polyclonal-anti-rabbit secondary antibodies (1:20,000; Thermo Fisher, Stockholm, Sweden) for 1 h at room temperature in TBS pH 7.6, 5% *w*/*v* non-fat milk and 0.05% *v*/*v* Tween 20. After washing, fluorescent bands were detected and quantified by Odyssey CLx and Image Studio software (LI-COR Biosciences, Lincoln, NE, USA). GFAP and β-actin, or S100β and β-actin protein levels were corrected to their brightest signal within each membrane and normalized to β-actin loading control corrected values. Values are given as fluorescence units.

### 2.4. Ussing Chamber Experiments with EGC Mediators

Ileal lamina propria tissues from six patients with CD median age 47 years (range 25–49, 3 men) and six non-IBD controls, median age 72 years (range 67–74, 2 men) were mounted in Ussing chambers as previously described [12]. After 40 min of equilibration [12], samples were collected to set baseline values. GSNO, 100 mM, (Sigma-Aldrich, Stockholm, Sweden), or 3 nM GDNF (Thermo Fisher, Stockholm, Sweden), or combination of both was added into three chambers respectively. Finally, three chambers served as vehicle control and Krebs buffer was added. Concentrations of GSNO and GDNF were based on our previous publication [19]. To study the effects of EGC mediators on paracellular permeability, 34 µCi/mL of the inert probe ^51^Chromium-EDTA (^51^Cr-EDTA) (Perkin Elmer, Boston, MA, USA), MW 384 Da, was added to all tissues on the mucosal side [20]. After 60 and 120 min, serosal samples were collected and ^51^Cr-EDTA was detected in a gamma-reader (1282 Compugamma, LKB, Bromma, Sweden). Permeability was calculated over time and given as P_app_ (apparent permeability coefficient; cm/s ×10^−6^).

### 2.5. Statistical Analysis

The data are presented as median and interquartile range (IQR). Statistical analysis was performed using GraphPad Prism version 9.1.2 (GraphPad Software, LLC). The outliers were identified using the ROUT test. The distribution of the data was tested for normality, using the D’Agostino-Pearson omnibus normality test. Comparisons between groups were performed using the Mann–Whitney U test and the Wilcoxon matched-pairs signed-rank test. The influence of patients’ characteristics on the results was tested using the Spearman correlation test.

## 3. Results

### 3.1. Increased Numbers of EGC^GFAP+^ in CD Patients Compared to Non-IBD Controls

**Figure 2:**
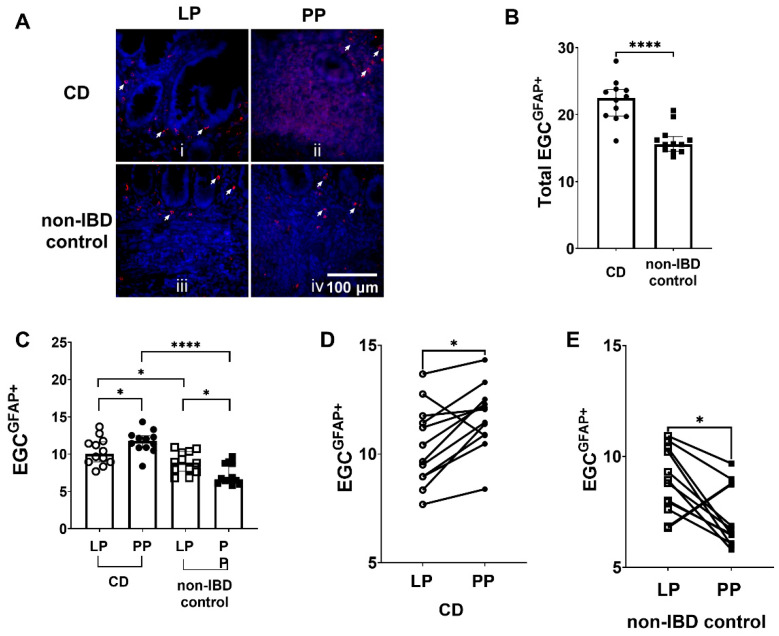
Enteric glial cells (EGC) expressing glial fibrillary acidic protein (EGC^GFAP+^) are more prominent in Crohn’s disease (CD). (**A**) Immunofluorescent staining of EGC^GFAP+^, i in lamina propria (LP) of CD patients, ii in Peyer’s patches (PP) of CD patients, iii in LP of non-inflammatory bowel disease (non-IBD) controls and iv in PP of non-IBD controls. The EGC^GFAP+^ are indicated by the arrows. Scale bar 100 μm. (**B**) Quantification of EGC^GFAP+^ in CD patients and non-IBD controls (**C**) The distribution of EGC^GFAP+^ in LP and PP of CD patients and non-IBD controls (**D**) The number of EGC^GFAP+^ was higher in PP of 11 out of the 12 CD patients analyzed, compared to LP (**E**) The number of EGC^GFAP+^ was lower in PP of 11 out of the 12 non-IBD controls analyzed, compared to LP. Data presented as median and interquartile range (IQR). Mann–Whitney U test was used for comparisons between groups and Wilcoxon matched-pairs signed-rank test for paired data, * *p* < 0.05, **** *p* < 0.0001.

The total number of EGC^GFAP+^ was quantified in immunostained sections from patients with CD and non-IBD controls (Figure 2A). There were significantly more (*p* < 0.0001) EGC^GFAP+^ in total (Peyer’s patches + lamina propria) in tissue sections from CD patients compared to non-IBD controls (Figure 2B).

### 3.2. Differences in EGC^GFAP+^ Distribution between Peyer’s Patches and the Lamina Propria in CD Patients and Non-IBD Controls, but Also between CD Patients and Non-IBD Groups

When separating the distribution of EGC^GFAP+^ into the Peyer’s patches and the lamina propria, results showed significantly more EGC^GFAP+^ in both the Peyer’s patches (*p* < 0.0001) and in the surrounding lamina propria (*p* < 0.05) in patients with CD patients compared to non-IBD controls (Figure 2C).

Later, we compared the numbers of EGC^GFAP+^ in the Peyer’s patches and the surrounding lamina propria in tissue from the same individual by immunofluorescent staining (Figure 2A, panels i and ii). Quantification of images showed significantly more (*p* < 0.05) EGC^GFAP+^ present in the Peyer’s patches compared to the lamina propria of CD patients (Figure 2C). Out of the 12 CD patients, 11 showed higher numbers of EGC^GFAP+^ in the Peyer’s patches compared to the lamina propria (Figure 2D).

In contrast to what we observed in CD patients, non-IBD samples revealed significantly decreased (*p* < 0.05) numbers of EGC^GFAP+^ in the Peyer’s patches compared to lamina propria (Figure 2A(iii,iv),C). Out of the 12 non-IBD patients, 11 showed lower numbers of EGC^GFAP+^ in the Peyer’s patches compared to the lamina propria (Figure 2E).

### 3.3. Western Blotting Confirmed the Differences in GFAP Expression between the Peyer’s Patches and Lamina Propria in CD Patients and Non-IBD Controls

**Figure 3:**
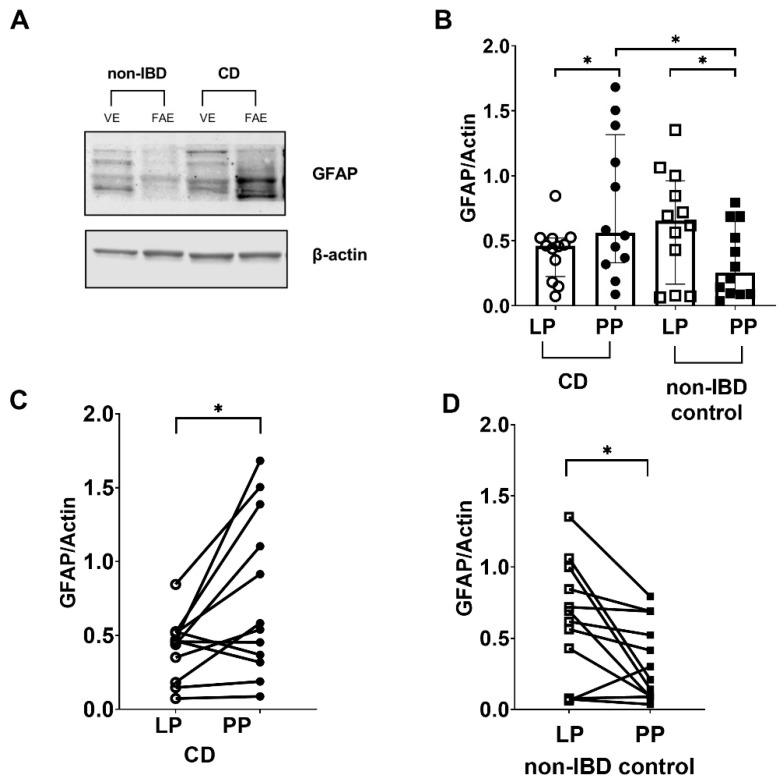
Glial fibrillary acidic protein (GFAP) levels are increased in tissue lysates of Crohn’s disease (CD) patients compared to non-inflammatory bowel disease (non-IBD) controls. (**A**) Representative Western blot image (converted into a black and white density blot) showing GFAP bands from one CD patient and one non-IBD control, respectively (**B**) Quantification of the bands after normalizing GFAP levels against the β-actin loading control (**C**) GFAP protein levels were higher in Peyer’s patches (PP) of 9 out of the 12 CD patients analyzed, compared to lamina propria (LP) (**D**) GFAP protein levels were lower in PP of 10 out of the 12 non-IBD controls analyzed, compared to LP. Data presented as median and IQR. Mann–Whitney U test was used for comparisons between groups and Wilcoxon matched-pairs signed-rank test for paired data, * *p* < 0.05.

We performed Western blot analysis (Figure 3A,B), to confirm the results from immunofluorescence. Comparison between the groups showed significantly more GFAP expression (*p* < 0.05) in the Peyer’s patches of CD patients when compared to non-IBD controls (Figure 3B), and less GFAP expression (*p* = 0.13) in lamina propria of CD patients when compared to non-IBD controls (Figure 3B).

Paired comparisons within the groups showed significantly increased GFAP protein expression (*p* < 0.05) in the Peyer’s patches compared to the lamina propria in CD patients (Figure 3B). Out of the 12 CD patients, nine showed increased expression of GFAP in the Peyer’s patches compared to the lamina propria (Figure 3C). In the non-IBD control group, GFAP expression was significantly decreased (*p* < 0.05) in Peyer’s patches compared to lamina propria (Figure 3B). Out of the 12 non-IBD patients, 10 showed decreased expression of GFAP in the Peyer’s patches compared to the lamina propria (Figure 3D).

### 3.4. Increased Numbers of EGC^S100β+^ in CD Patients Compared to Non-IBD Controls

**Figure 4:**
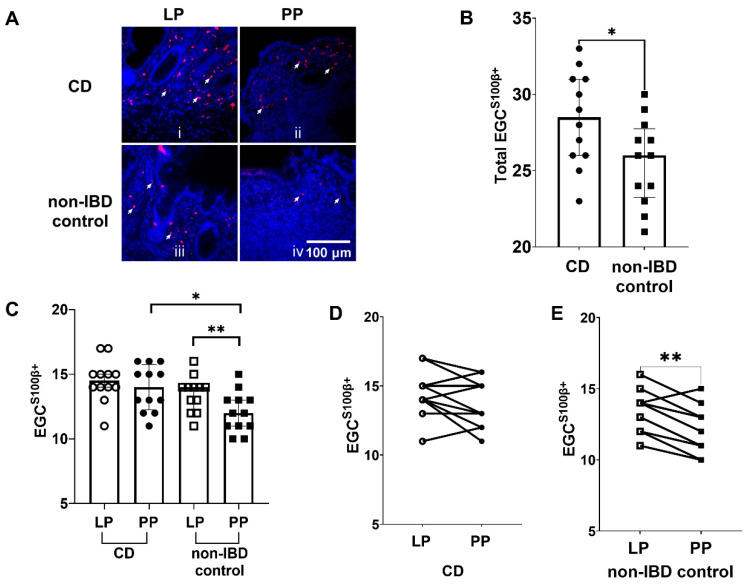
Enteric glial cells (EGC) expressing S100 calcium-binding protein β (EGC^S100β+^) are more prominent in Crohn’s disease (CD) patients. (**A**) Representative immunofluorescent images of EGC^S100β+^ in i the lamina propria (LP) of CD patients, ii Peyer’s patches (PP) from CD patients, iii LP of non-inflammatory bowel disease (non-IBD) controls and iv in PP of non-IBD controls. Arrows indicate EGC^S100β+^ cells. Scale bar 100 μm. (**B**) Quantification of EGC^S100β+^ in CD patients and non-IBD controls (**C**) The distribution of EGC^S100β+^ in LP and PP of CD patients and non-IBD controls (**D**) The number of EGC^S100β+^ was higher in PP of 4 out of the 12 CD patients analyzed, compared to LP (**E**) The number of EGC^S100β+^ was lower in PP of 11 out of the 12 non-IBD controls analyzed, compared to LP. Data presented as median and IQR. Mann–Whitney U test was used for comparisons between groups and Wilcoxon matched-pairs signed-rank test for paired data, * *p* < 0.05, ** *p* < 0.01.

Like for GFAP, we assessed the distribution of EGC in Peyer’s patches through immunofluorescent staining using S100β as a marker (Figure 4A, panels i and ii). The quantification of the images showed significantly more (*p* < 0.05) EGC^S100β+^ in total (Peyer’s patches + lamina propria) of CD patients compared to non-IBD controls (Figure 4B).

### 3.5. Higher Numbers of EGC^S100β+^ in the Peyer’s Patches of CD Patients Compared to Non-IBD Controls, and Lower Numbers of EGC^S100β+^ in Peyer’s Patches Compared to Lamina Propria in Non-IBD Controls

As for EGC^GFAP+^, we also observed differences in the number of EGC^S100β+^ between the two groups (Figure 4C). In the Peyer’s patches, we observed significantly more (*p* < 0.05) EGC^S100β+^ in CD patients when compared to non-IBD controls (Figure 4C). In the lamina propria, however, we did not observe significant differences between the groups (Figure 4C).

Paired comparison between the numbers of EGC^S100β+^ in the Peyer’s patches and lamina propria of CD patients did not show any significant differences (Figure 4C). Out of the 12 CD patients, four showed increased numbers of EGC^S100β+^ in the Peyer’s patches compared to the lamina propria (Figure 4D).

In the non-IBD controls, we observed significantly lower numbers (*p* < 0.01) of EGC^S100β+^ in the Peyer’s patches, compared to the lamina propria, (Figure 4C). Out of the 12 non-IBD patients, 11 showed decreased numbers of EGC^S100β+^ in the Peyer’s patches compared to the lamina propria (Figure 4E).

### 3.6. Differences in S100β Expression between Peyer’s Patches and Lamina Propria in CD Patients and Non-IBD Controls by Western Blotting

**Figure 5:**
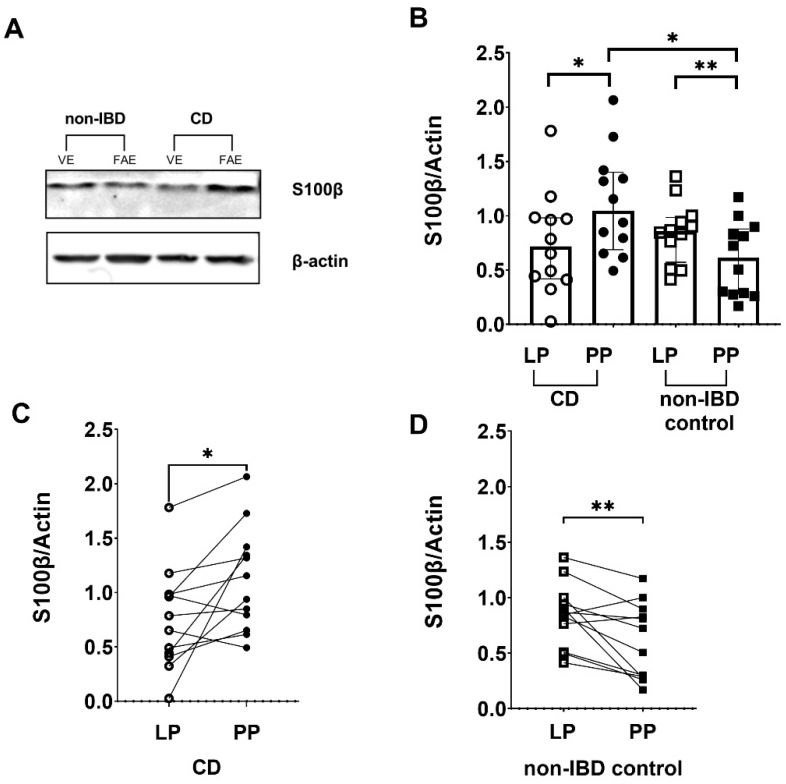
S100 calcium-binding protein β (S100β) levels are increased in tissue lysates of Crohn’s disease (CD) patients compared to non- inflammatory bowel disease (non-IBD) controls (**A**) Representative Western blot image (converted into a black and white density blot) showing S100β bands from one CD patient and one non-IBD control, respectively (**B**) Quantification of the bands after normalizing S100β levels against the β-actin loading control (**C**) S100β protein levels increased in Peyer’s patches (PP) of 10 out of the 12 CD patients analyzed, compared to lamina propria (LP) (**D**) S100β protein levels decreased in PP of 10 out of the 12 non-IBD controls analyzed, compared to LP. Data presented as median and interquartile range. Mann–Whitney U test was used for comparisons between groups and Wilcoxon matched-pairs signed-rank test for paired data, * *p* < 0.05, ** *p* < 0.01.

To confirm our observation from immunofluorescent staining of S100β, we performed Western blot (Figure 5A,B). Comparison between the groups showed equal levels of S100β expression in CD patients and non-IBD controls in the lamina propria (Figure 5B); however, in the Peyer’s patches the expression was significantly higher in CD patients (*p* < 0.05) (Figure 5B).

Paired comparisons within the CD patients group showed significantly increased S100β protein expression (*p* < 0.05) in the Peyer’s patches compared to the lamina propria (Figure 5C). Out of the 12 CD patients, 10 showed increased expression of S100β in the Peyer’s patches compared to the lamina propria (Figure 5C). In the non-IBD group, S100β expression was significantly decreased (*p* < 0.01) in the Peyer’s patches compared to lamina propria (Figure 5D). Out of the 12 non-IBD patients, 10 showed decreased expression of S100β in the Peyer’s patches compared to the lamina propria (Figure 5D).

### 3.7. Increased Paracellular Permeability by EGC Mediators in CD Patients While Decrease in Controls

**Figure 6:**
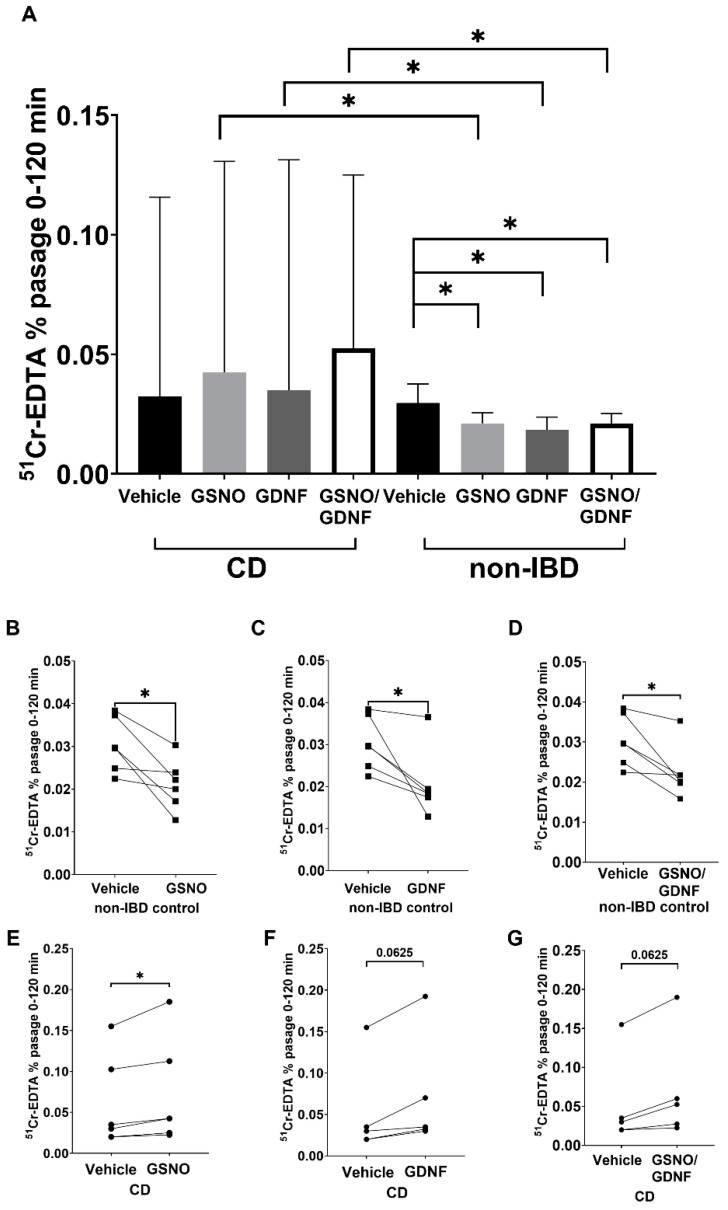
Enteric glial cells (EGC) mediators increase epithelial barrier function in non-inflammatory bowel disease (non-IBD) controls while decrease it in Crohn’s disease (CD) patients. (**A**) Paracellular permeability of ^51^Chromium-EDTA (^51^Cr^-^EDTA) through the epithelium of non-IBD controls and in CD patients in the presence of vehicle or EGC mediator S-nitroglutathione (GSNO), glial-derived neurotrophic factor (GDNF), or the combination of both (GSNO/GDNF). (**B**) ^51^Cr^-^EDTA permeability through the epithelium of non-IBD controls with and without stimulation with GSNO. (**C**) ^51^Cr^-^EDTA permeability through the epithelium of non-IBD controls with and without stimulation with GDNF. (**D**) ^51^Cr^-^EDTA permeability through the epithelium of non-IBD controls with and without stimulation with GSNO/GDNF. (**E**) ^51^Cr^-^EDTA permeability through the epithelium of CD patients with and without stimulation with GSNO. (**F**) ^51^Cr^-^EDTA permeability through the epithelium of CD patients with and without stimulation with GDNF. (**G**) ^51^Cr^-^EDTA permeability through the epithelium of CD patients with and without stimulation with GSNO/GDNF. Mann–Whitney U test was used for comparisons between groups and Wilcoxon matched-pairs signed-rank test for paired data, * *p* < 0.05.

After 120 min in Ussing chambers, samples were collected from the serosal side for measurement of ^51^Cr-EDTA. We observed a significant increase (*p* < 0.05) in the passage of ^51^Cr-EDTA through CD patients’ tissues stimulated with GSNO, GDNF, and GSNO/GDNF, respectively, compared to non-IBD control (Figure 6A). Furthermore, we observed a significant decrease (*p* < 0.05) in the passage of ^51^Cr-EDTA in non-IBD control tissues stimulated with GSNO (Figure 6B), GDNF (Figure 6C), and GSNO/GDNF (Figure 6D) which is in line with our previous findings in biopsies from healthy controls [19]. Interestingly, the opposite pattern was seen for CD patients, where all stimuli resulted in an increased passage of ^51^Cr-EDTA through tissues compared to vehicle (Figure 6E–G). The increase was significant for GSNO (*p* < 0.05) (Figure 6E) but not significant for GDNF (Figure 6F) and GSNO/GDNF (*p* = 0.0625) (Figure 6G); however, this was most likely due to the lower number of CD patients included (*n* = 5) for these stimuli.

### 3.8. No Effect on the Results by Patient Characteristics

There was no significant influence on the results within the groups either by age, sex, anti-inflammatory medication, indication for surgery, primary/recurrent surgery or p-CRP. Moreover, there was no significant impact of age between the groups.

## 4. Discussion

We assessed, for the first time, the distribution of EGC in the Peyer’s patches and compared it between patients with CD and non-IBD controls. By immunofluorescent staining, we observed more EGC in the Peyer’s patches of CD patients compared to non-IBD, with both GFAP and S100β as markers. Further analysis of the EGC distribution showed more EGC in the Peyer’s patches of CD patients compared to the surrounding lamina propria but also compared to the Peyer’s patches of non-IBD controls. These observations were confirmed by western immunoblotting, of the Peyer’s patches and the lamina propria, where we observed significant differences in expression of both GFAP and S100β. Finally, we assessed the effect of EGC mediators on barrier function ex vivo using Ussing chambers. We confirmed our previous findings [19] of a decreased paracellular permeability by ECG mediators in non-IBD controls; however, results showed an increased paracellular permeability by EGC mediators in CD patients. This is a novel finding which points to that EGC might have diverse functions during inflammation and health [21].

One of the observations presented in this study is the higher number of EGC in CD patients compared to non-IBD controls. The participants in the non-IBD group were older (median age 73 years) than the patients in the CD group (median age 43 years). Phillips et al. [22] showed that age may affect the number of EGC present in the ileum of rats. However, a reduction in the number of EGC has not been confirmed in humans. It has actually been proposed that EGC may proliferate as a result of a reduction in the number of neurons in the ENS in rats [23], but neither this has been confirmed in humans. In the case of CD, studies have shown that the enteric nerves might be injured [24], but the number of enteric neurons is not different between CD patients and non-IBD controls [25]. Therefore, we believe that the higher number of EGC observed in CD patients is most likely associated to the inflammation and might be an important contributing factor to the pathogenesis of the disease.

It was previously suggested that the inflammation in CD starts at the FAE covering the Peyer’s patches [6]. Although Peyer’s patches have been implicated in CD pathogenesis, there are, to our knowledge, no published studies on EGC distribution in the Peyer’s patches of CD patients. There are very few studies on EGC and CD overall. However, in a study by Villanacci et al. [25], immunohistochemistry was used to identify the distribution of EGC by S100β staining in ileal resected tissue from patients with CD and non-IBD controls. The authors observed that the number of EGC^S100β+^ increased in involved areas compared to non-involved areas of CD patients, something that was not investigated in our study where only non-inflamed tissues were studied. They further reported no significant difference in EGC^S100β+^ numbers between CD patients’ ileum and the ileum of controls, which is in line with our findings showing equal numbers of EGC^S100β+^ in the lamina propria of CD patients and controls by immunofluorescence. In terms of assessing S100β expressions in ileal tissue by Western blot, we cannot compare our findings to others, since to our knowledge, no published studies are assessing the S100β levels by Western blot, neither in colonic nor in ileal tissue of CD patients. We found it challenging to measure S100β protein expression through Western blot, because S100β was easily degraded, probably because of the freeze-thaw cycles of the biopsy lysates.

Most of the studies using GFAP as a marker for EGC used colon rather than ileal tissue; however, these studies are also conflicting. Boyen et al. [26] reported an increased GFAP expression by immunohistochemistry and western blotting in inflamed colonic biopsies compared to non-inflamed in patients with CD. At the same time, authors observed lower levels, but not significantly lower, of GFAP in non-inflamed colon from CD patients compared to the colon of controls [26]. Cornet et al. [27], using western blotting and ELISA, could not show differences in GFAP levels between inflamed CD areas, non-inflamed CD areas, and non-IBD control. The results presented in the current study originate from pooled data from ileal and colonic tissue, preventing us from directly comparing our observations to the findings from the cited study. Steinkamp et al. [28] showed an increased number of EGC^GFAP+^ in the colon of CD patients when compared to non-IBD controls. Due to the different approaches between the previous studies or focus of the colon rather than the ileum, it is difficult to compare our results to previously published data. We for the first time quantified EGC expressing GFAP and S100β by immunofluorescence and western blotting in the ileum of CD patients and non-IBD controls, and in addition, we distinguished between the distribution in the Peyer’s patches and the surrounding lamina propria. When we compared between EGC^GFAP+^ and EGC^S100β+^, we observed differences in the numbers. Due to the methods used, single rather than double staining, we cannot determine if GFAP and S100β are expressed in the same or different EGC and further work is needed to address this issue. Previous work in mice showed that the two markers are expressed in different EGC and do not always overlap [29]. This novel observation is supported by previous reports showing that GFAP expression can be associated with pro-inflammatory markers such as IL-1β [30]. S100β is reported to act in a dose-dependent manner in the central nervous system. In low doses, S100β promotes neuron survival, while in high doses it promotes apoptosis [31,32]. In addition, in damaged cardiomyocytes, S100β is reported to activate the NF-kB response [31,32]. To our knowledge, the role of S100β in the gut is still unclear and we can only speculate that it has similar functions as in other systems in the human body.

Several subgroups of EGC were recently described [16,29,33] and future studies are needed to elucidate this in CD patients. One of the questions, relevant to this study, is if the different EGC subpopulations secrete the same mediators and control the intestinal epithelial barrier in the same or different manner. It is generally accepted that EGC mediators, such as GDNF, improve the epithelial integrity [34,35], promote cell to cell and cell to matrix adhesion [36], and reduce paracellular permeability in non-IBD controls as we showed in this study and as we previously reported [19,37]. However, our current results of the effect of EGC mediators on ileal tissues from CD patients mounted in Ussing chambers, showed an increased permeability. Several factors may contribute to this interesting observation. First, very little is known about the different subtypes of EGC, their role in health and disease as well as their response upon stimulation. Our findings indicate that EGC mediators in CD patients have an opposite effect compared to health. It has been reported that the production of GDNF has a protective role in EGC survival [28], which probably is not detectable in the described ex vivo experiments due to relatively short experimental time. Apart from the effect of GDNF on the survival of EGC, very little has, to our knowledge, been reported about potential feedback loops and pathways that can be activated within EGC after stimulation with GDNF. In addition, the levels of GDNF present in the intestinal tissues of CD patients were reported to be lower compared to non-IBD controls [35].A recent study has shown that GDNF prevents the degranulation of mast cells in the colon of dextran sulfate sodium-induced colitis in rats, and at the same time, it results in decreased production of pro-inflammatory cytokines in an in vitro cell model of RBL-2H3 cells and rats [38].These observations from cell models are opposite to what we observed in human biopsies ex vivo. The mechanisms that explain our observations, compared to what we would expect from previously published data, can involve several factors. First, the external supplementation of the biopsies with GDNF, GSNO, or the combination of both may have activated an unknown feedback loop in EGC that is promoting the production of pro-inflammatory cytokines that can decrease the epithelial barrier function. Additionally, the human intestine includes the epithelium and the immune cells residing in the lamina propria, such as eosinophils, mast cells, and macrophages, and the Peyer’s patches that are rich in antigen-presenting cells, T and B cells. We previously reported an increased number of mast cells in the Peyer´s patches of CD patients and an increased number of mast cells is associated with the neuropeptide VIP [10]. The questions that arise are how do GDNF and GSNO affect the production of VIP, and what is the role of VIP in the maintaining of the epithelial barrier during health and inflammation. We know, from our previous studies that VIP increases the bacterial passage in the human Peyer’s patches [39] and that the bacterial passage is increased in Peyer’s patches of CD patients compared to non-IBD controls [13,40]. It has become evident that VIP has both, anti-inflammatory [41,42] and pro-inflammatory effects [43,44]. We recently showed [19] that VIP induces an increased expression of GFAP in an EGC cell line, indicating a direct activating effect on EGC by VIP. Furthermore, it has been shown that lipopolysaccharide promotes EGC to produce IL-1β [45], and IL-1β is known to increase intestinal permeability [46]. Moreover, when infecting EGC in vitro with living *Salmonella typhimurium*, the GFAP expression significantly increased as well as the release of S100β from the EGC [19]. Taking these facts together, we speculate that the increased translocation of bacteria over the Peyer’s patches in CD patients can lead to an over-stimulation of EGC which might induce up-regulation of the GFAP and S100β expression. Furthermore, the up-regulation might be due to the increased number of mast cells secreting VIP in the Peyer’s patches which in turn activates the EGC, and hypothetically this may explain why we find more EGC^GFAP+^ and EGC^S100β+^ in Peyer’s patches of CD patients compared to non-IBD controls. Further work is needed to identify the exact mechanisms behind the higher number of EGC in the Peyer’s patches of CD patients and effects of EGC mediators on the production of VIP, and their effects on other immune cells and the intestinal barrier.

## 5. Conclusions

In conclusion, we showed a higher number of EGC in the Peyer’s patches of CD patients compared to the surrounding lamina propria and compared to non-IBD controls. In addition, EGC mediators increased the permeability in CD patients while having protecting effects on the barrier in non-IBD, suggesting a harmful impact on the barrier which might contribute to the pathophysiology of the disease.

## Figures and Tables

**Table 1:** Characteristics of the 20 patients with Crohn’s disease included in the study.

Age (y)	Sex	Anti-Inflammatory Medication	Indication for Surgery	Primary or Recurrent Surgery	Montreal Classification	Pre-op p-CRP
61	M	None	Stricture	Primary	A2L3B2	13
63	M	None	Stricture, abscess	Recurrent	A2L3B3	<10
20	M	Azathioprine	Stricture, fistula	Primary	A2L1B3	<10
38	F	None	Stricture	Primary	A2L1B2	10
38	F	None	Stricture	Primary	A2L3B2p	<10
49	F	None	Stricture, abscess	Primary	A3L1B2	<10
50	M	Azathioprine, infliximab	Stricture	Primary	A2L1B2	<10
25	F	Azathioprine	Stricture	Recurrent	A1L1B3	<10
49	M	None	Stricture, abscess	Recurrent	A2L1B3	26
46	M	None	Stricture	Primary	A2L1B2	<10
43	F	None	Stricture	Primary	A2L3B2	42
49	M	None	Stricture	Recurrent	A2L1B3	<10
49	F	Ustekinumab	Stricture	Recurrent	A2L3B2	<10
55	M	Azathioprine, infliximab	Stricture	Recurrent	A2L1B2	<10
29	F	None	Fistulas	Recurrent	A1L1B3	<10
29	M	Thiopurine, adalimumab	Fistula	Recurrent	A2L3B3p	<10
43	M	Mesalazine, infliximab	Fistula	Primary	A2L3B3	<10
27	M	None	Stricture, fistula, abscess	Recurrent	A1L3B3	19
21	M	None	Stricture	Recurrent	A2L1B3	<10
17	F	Budesonide, azathioprine, infliximab	Stricture	Primary	A1L3B2	<10

NOTE: Age at diagnosis: A1: <16, A2: 16–40, A3: >40, Location of disease: L1: ileal, L3: ileocolonic, Behavior of disease B2: strictures, B3: perforations., p: perianal disease, Pre-op p-CRP= pre-operative plasma-C-reactive protein.

## Data Availability

Not applicable.
